# Chronic Hyperplastic Candidiasis—An Adverse Event of Secukinumab in the Oral Cavity: A Case Report and Literature Review

**DOI:** 10.3390/diseases13080243

**Published:** 2025-08-03

**Authors:** Ana Glavina, Bruno Špiljak, Merica Glavina Durdov, Ivan Milić, Marija Ana Perko, Dora Mešin Delić, Liborija Lugović-Mihić

**Affiliations:** 1 Department of Dental Medicine, University Hospital of Split, 21000 Split, Croatia; 2 Department of Oral Medicine, Study of Dental Medicine, School of Medicine, University of Split, 21000 Split, Croatia; marijaperko99@gmail.com (M.A.P.); d.mesin.st@gmail.com (D.M.D.); 3 Department of Oral Medicine, University of Zagreb School of Dental Medicine, 10000 Zagreb, Croatia; 4 Department of Pathology, University Hospital Split, 21000 Split, Croatia; merigdst@yahoo.co.uk; 5 School of Medicine, University of Split, 21000 Split, Croatia; 6 Department of Urology, University Hospital of Split, 21000 Split, Croatia; ivan.milic5@gmail.com; 7 Department of Dermatovenereology, University Hospital Center Sestre Milosrdnice, 10000 Zagreb, Croatia; liborija@gmail.com; 8 School of Dental Medicine, University of Zagreb, 10000 Zagreb, Croatia

**Keywords:** anti-interlekin-17A, chronic hyperplastic candidiasis, oral adverse event, psoriasis, psoriatic arthritis, secukinumab

## Abstract

Secukinumab (SEC) is a recombinant, fully human monoclonal antibody that is selective for interleukin-17A (IL-17A). SEC may increase the risk of developing infections such as oral herpes and oral candidiasis. The aim of this case report and literature review was to describe chronic hyperplastic candidiasis (CHC) in a patient with psoriasis (PsO) and psoriatic arthritis (PsA) treated with SEC. CHC is a rare and atypical clinical entity. A definitive diagnosis requires biopsy of the oral mucosa for histopathological diagnosis (PHD). The differential diagnosis includes hairy tongue, hairy leukoplakia, oral lichen planus (OLP), oral lichenoid reaction (OLR), leukoplakia, frictional keratosis, morsication, oral psoriasis, syphilis, and oral lesions associated with coronavirus disease (COVID-19). In addition to the usual factors (xerostomia, smoking, antibiotics, vitamin deficiency, immunosuppression, comorbidities), the new biological therapies/immunotherapies are a predisposing factor for oral candidiasis. The therapeutic approach must be multidisciplinary and in consultation with a clinical immunologist. Dentists and specialists (oral medicine, dermatologists, rheumatologists) must be familiar with the oral adverse events of the new biological therapies. Simultaneous monitoring of patients by clinical immunology and oral medicine specialists is crucial for timely diagnosis and therapeutic intervention to avoid possible adverse events and improve quality of life (QoL).

## 1. Introduction

According to the World Health Organisation (WHO) definition, an adverse event is a reaction to a medicinal product that is harmful and unintended and that occurs at doses normally used in humans for the prophylaxis, diagnosis, or treatment of disease or to modify physiological functions. It may occur immediately after taking the drug or after several years of continuous use. A detailed medical history (in addition to clinical-oral findings) is a key factor for an accurate diagnosis [1]. Secukinumab (SEC, Cosentyx^®^, Novartis Pharmaceuticals, East Hanover, NJ, USA) is a recombinant, fully human monoclonal antibody that is selective for interleukin-17A (IL-17A). The US Food and Drug Administration (FDA) approved it for the treatment of psoriasis (PsO) in 2015 [2]. It is indicated for the treatment of moderate to severe plaque PsO, active psoriatic arthritis (PsA), or ankylosing spondylitis (AS) [3]. SEC is usually administered subcutaneously (s.c.) at a dose of 300 mg (two injections of 150 mg) at 0, 1, 2, 3, and 4 weeks, with treatment continuing at the same dose once a month [4]. SEC prevents the binding of IL-17A to receptors found in many cells, including keratinocytes, and thus contributes to better control of autoimmune and inflammatory diseases by reducing the proinflammatory milieu [5,6,7,8,9].

SEC may increase the risk of developing infections that are mainly mild or moderate. Serious infections in patients treated with SEC are rare with a prevalence of 0.14% [4]. IL-17A plays a key role in protecting the host from opportunistic fungal infections [10]. The most common oral infections are oral herpes and oral candidiasis. Infection rates in clinical studies are similar for PsO, PsA, and AS [9]. The first case of chronic hyperplastic candidiasis (CHC) as an oral adverse event of SEC was reported in 2021. Cases of chronic mucocutaneous candidiasis (CMC) as an oral adverse event of SEC have not been documented. Urticaria and anaphylactic reactions have been reported very rarely in connection with the use of SEC [4]. Patients with chronic mucocutaneous disease who have mutations in interleukin-17 (IL-17)-related genes also have persistent or recurrent *Candida* infections [11].

IL-17 is an important immune factor in the pathogenesis of autoimmune diseases, which is mainly produced by T lymphocytes, in particular, Th17 [12,13]. The IL-17 family consists of six members from IL-17A to IL-17F [13]. In addition to the aforementioned PsO, PsA, and AS, IL-17 plays a role in the pathogenesis of numerous autoinflammatory diseases such as rheumatoid arthritis (RA), inflammatory bowel disease (IBD), and multiple sclerosis (MS) [12,14]. PsO is a chronic autoinflammatory disease in which proinflammatory cytokines [IL-17A dominant, IL-17C, IL-17F, tumour necrosis factor-α (TNF-α)], chemokines, and prostaglandins promote increased proliferation of epidermal cells [15,16,17]. In recent years, PsO has been successfully treated with an increasing number of new biologic drugs such as SEC, ixekizumab (IL-17A), brodalumab (IL-17RA), and bimekizumab (IL-17A and IL-17F) [18,19]. They target one or more IL-17 receptors and predispose patients to the development of various forms of candidiasis [20].

CHC is a rare and atypical clinical entity and represents a diagnostic challenge. Furthermore, by systematically reviewing the literature, we aim to contribute to the current state of knowledge on whether oral adverse events associated with SEC are drug-related (drug-specific) or class-related. We wanted to draw the attention of general dentists and oral medicine specialists to this clinical entity, the early detection of which is crucial for successful treatment and improvement in the patient’s quality of life (QoL). The objective of this case report and literature review was to describe CHC in a patient with PsO and PsA treated with SEC.

## 2. Materials and Methods

A structured search of relevant scientific databases was performed for this case report and the literature review: PubMed (MEDLINE), Embase, Scopus, Web of Science, and Google Scholar. All available articles from January 2015 to July 2025 were considered, as SEC was approved for clinical use in 2015. The search was performed using combinations of the following keywords and Boolean combinations: “ecukinumab” OR “anti-IL-17” OR “IL-17”, “oral cavity” OR “oral lesions” OR “oral” OR “mouth” OR “tongue”, “chronic hyperplastic candidiasis” OR “candidal leukoplakia”, “oral candidiasis” OR “oral fungal infection”, “adverse event” OR “side effect” OR “oral complication”, “case report” OR “case reports” OR “case study”. Case reports, case series, and reviews describing individual cases of patients treated with SEC in English and articles with English abstracts were included without geographical restriction. Studies on adults with oral lesions documented clinically and by histopathological diagnosis (PHD) were included. Articles on other forms of fungal infections (skin, genitalia), adverse events of other biologics, articles without available abstracts, and non-peer-reviewed articles were excluded. Animal and in vitro studies, systematic reviews, and meta-analyses were also excluded. Two authors (A.G. and B.Š.) independently reviewed the titles and abstracts. The included articles were then analysed in full text, including clinical presentation, PHD, disease progression in relation to SEC, and outcome after therapy.

## 3. Results—Case Report

A 74-year-old male patient came to his first specialist examination at the oral medicine clinic, Department of Dental Medicine, Department of Maxillofacial Surgery, University Hospital Centre Split, Split, Croatia, in February 2024. He subjectively complained of a “burning tongue”, especially when eating. He stated that the first problems with the tongue (burning sensation) occurred 3 months after the start of SEC treatment. He then underwent an examination by another oral medicine specialist (in the same institution), whose final diagnosis was a geographic and fissured tongue. The family history was non-contributory. The medical history included PsO and PsA, percutaneous coronary intervention (PCI) 10 years ago, gastro-oesophageal reflux disease (GERD), and indolent B non-Hodgkin’s lymphoma (NHL) confined to the left pleura (with no B symptoms and no evidence of disease spread). He has been taking oral anticoagulants and SEC s.c. once a month for 36 months. He denied drug allergies and was a non-smoker.

**Figure 1:**
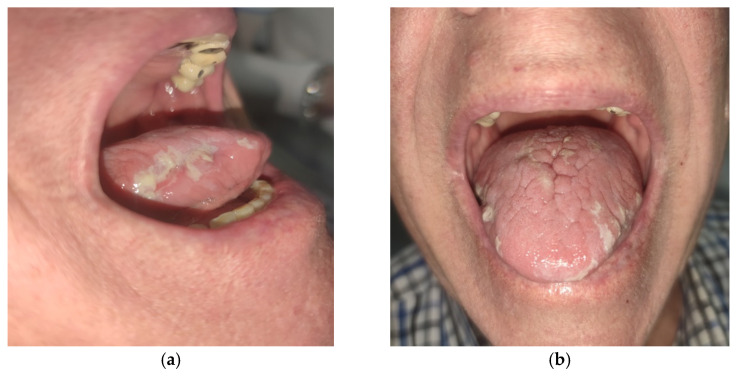
Keratotic lesions of chronic hyperplastic candidiasis (CHC) on the lateral sides (**a**) and dorsum (**b**) of the tongue.

Clinically, there were extensive thick keratotic lesions on the mucosa of the tongue—the mucosa of the dorsum of the tongue (middle third) and the lateral side of the tongue on both sides (anterior third) (Figure 1). Clinically, the lesions were white and keratotic, could not be scratched out, and were bilaterally and symmetrically distributed. In addition, there were irregularly shaped erosions on the lateral side of the tongue on both sides with a diameter of 7 mm (anterior third) (the patient stated that he had “plucked” the white lesions). There was a fissured tongue on the mucosa of the dorsum of the tongue. The rest of the oral mucosa was normal. He denied changes to other mucous membranes and the skin.

**Figure 2:**
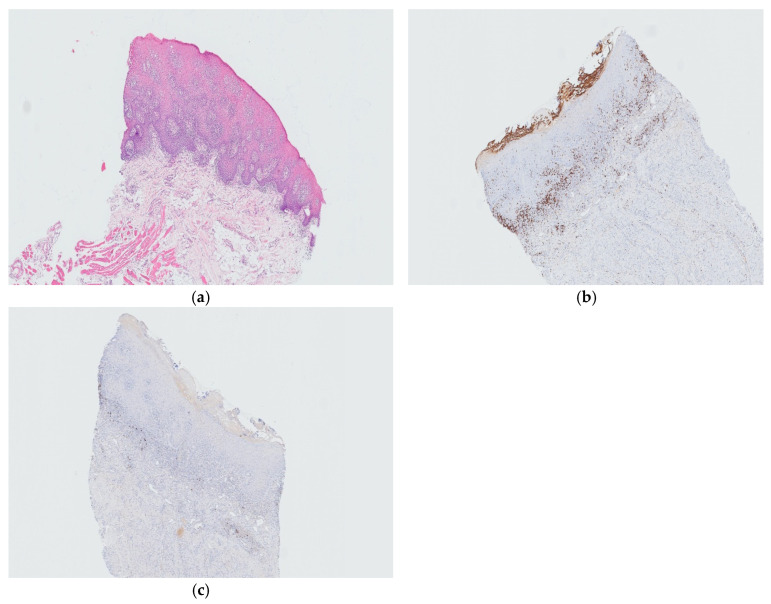
Thick squamous epithelium with marked acanthosis (**a**) and a dense infiltrate of CD8+T lymphocytes (**b**) and rare scattered CD20+ B lymphocytes (**c**) in the subepithelial stroma (40×).

Immunological findings showed increased values for IgA (4.65; reference values 0.70–4.00 g/L) and beta-globulins (10.0; reference values 6.00–9.40 g/L). The antinuclear antibodies (ANA) were negative, while the extractable nuclear antigens (ENAs) were non-contributory. The enzyme-linked immunosorbent assay (ELISA) for anti-BP Ag 1 and 2 and anti-Dsg 1 and 3 was negative. A biopsy of the lateral side of the tongue was performed for PHD and direct immunofluorescence (DIF). Histological examination revealed parakeratosis and acanthosis in the stratified squamous epithelium with bacterial colonies on the surface. Apoptotic bodies and vacuolated basal keratinocytes were noted. The underlying stroma showed a dense mononuclear infiltrate with scattered neutrophils (focally distributed). Immunohistochemically, these were T lymphocytes, predominantly of the helper phenotype (CD4+) and less of the cytotoxic phenotype (CD8+), while the infiltration of B lymphocytes (CD20+) was sparse. Lymphocytes were also found intraepithelially. The culture of the oral lesions for *Candida* was negative. Histochemical Periodic acid-Schiff (PAS) staining also revealed no fungal spores or hyphae. Although the KOH test (potassium hydroxide test) is a rapid and simple diagnostic method, we did not use it to detect fungal spores and hyphae, as we used much more sensitive diagnostic methods (PAS staining and culture). The immunohistochemical expression of p16 was negative in the epithelial cells. In situ hybridisation for Epstein–Barr virus (EBV)-encoded small RNA (EBER) was negative with a positive external control (the presence of EBV was not detected). Bundles of skeletal muscle and adipose tissue were present at the base (Figure 2). DIF showed no immune deposits of IgA, IgG, IgM, C3, C4, and C1q at the dermo-epidermal junction or intraepidermally. The pathological findings corresponded to a chronic inflammatory mucosal change with a lichenoid appearance, possibly caused by the drug—with the recommendation of a clinical–pathological correlation.

**Figure 3:**
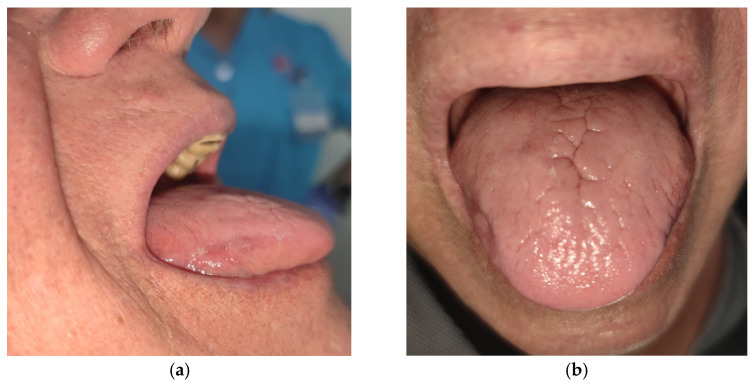
Disappearance of the lesions on the lateral sides (discrete residues visible) (**a**) and on the dorsum of the tongue (**b**) after 6 weeks of local and oral systemic antifungal therapy.

A clinical immunologist was consulted who, based on the medical documentation and clinical picture, decided to exclude SEC. Due to the class effect of IL-17A inhibitors, guselkumab—an interleukin 23 (IL-23) inhibitor—was added to the therapy. The patient was prescribed oral systemic therapy with fluconazole (150 mg 1× daily) and local therapy—hexetidine antiseptic (3× daily) and nystatin drops (30 drops 3× daily, shaking the whole mouth for 3 min and then swallowing—with the recommendation to shake the bottle before use)—for 6 weeks (with follow-ups every 2 weeks), which resulted in complete disappearance of the lesions (Figure 3).

## 4. Results—Literature Review

**Figure 4:**
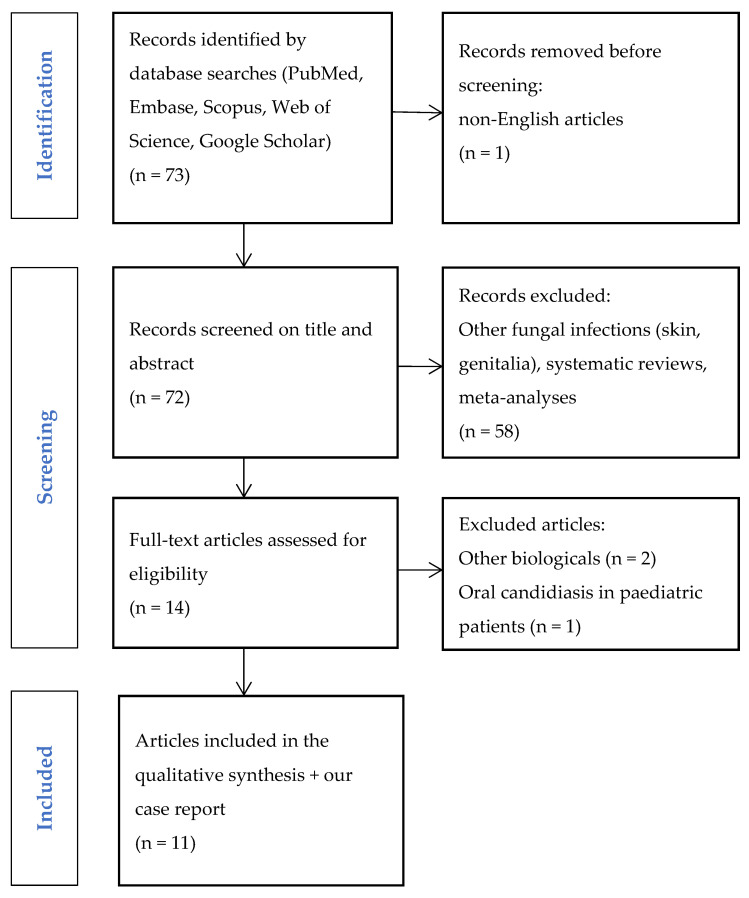
Study selection.

A systematic literature search was conducted to identify published cases of oral lesions associated with SEC. The search was conducted using a combination of controlled terms (MeSH, EMTREE) and free keywords related to SEC, oral cavity, and oral lesions. The search was filtered by human studies, English language, and case reports or case series. A total of 73 articles were found, one of which was removed because it was not written in English. After reviewing the titles and abstracts of 72 articles, 58 articles were excluded because they did not describe oral lesions in the context of SEC, systematic reviews, and meta-analyses. A comprehensive review of the remaining 14 articles included 11 relevant cases from the literature (our own case report was also included in this review). The selection process is illustrated in the PRISMA flow diagram (Figure 4) and the results summarised by database are shown in Table 1.

## 5. Discussion

CHC as an oral adverse event of SEC was first described in 2021 (4). The definitive diagnosis of CHC as an oral adverse event of SEC is supported by the fact that it occurred after the start of SEC (after 3 months, which is consistent with the systematic literature data) and completely disappeared after local and oral systemic antifungal therapy. The clinical appearance of the lesion also favoured CHC. The localisation of the mucosa of the dorsum of the tongue (middle third) and the lateral sides of the tongue on both sides (anterior third) were indicative of CHC, in contrast to hairy leukoplakia, whose lesions are mainly located on the lateral sides of the tongue (posterior third). Our patient had no other predisposing factors for the development of oral candidiasis (hyposalivation, smoking, medication), except for advanced age, which indicates a direct aetiological link with SEC. He only takes oral anticoagulants, which do not have dry mouth as a side effect (which was also confirmed by objective sialometric measurements). The clinical appearance of the lesions—extensive thick keratotic lesions, bilaterally and symmetrically distributed, which could not be scratched out—suggests an infectious, reactive, or autoimmune aetiology [21].

Histologically, a parakeratotic and acanthotic multilayered squamous epithelium was found, a microscopic feature that is non-specific but indicative of CHC. In the underlying stroma, a dense mononuclear infiltrate of T lymphocytes was found, focally with some neutrophil granulocytes, which did not show the typical band-like distribution seen in oral lichen planus (OLP). The histopathological findings had several rare and confusing elements that made the final diagnosis of CHC quite difficult. Firstly, some apoptotic bodies were found intraepidermally and vacuolated keratinocytes basally. Apoptotic bodies are rarely seen in CHC and are a non-specific sign. Vacuolised keratinocytes are also a non-specific sign and may be due to the local toxic effect of *Candida* metabolites or reactive changes. Apoptosis and vacuolisation are secondary and non-specific features of CHC. However, their presence may include the following differential diagnoses: lupus erythematosus (LE) (DIF excluded), oral lichenoid drug reaction (OLDR) (excluded due to the unilateral distribution of OLDR lesions, i.e., the bilateral and symmetrical distribution of the CHC lesions), chronic graft versus host disease (cGVHD) (excluded by history), and erythema exudativum multiforme (EEM) (excluded by history due to the acute onset of the EEM lesions, i.e., the chronic nature of the CHC lesions). Secondly, neither histochemical PAS staining nor culture revealed fungal spores or hyphae. Such a finding is rare and possible for several reasons: (1) the biopsy specimen did not contain spores/hyphae; (2) treatment with antifungals or oral antiseptics before the biopsy; (3) technical reasons in the laboratory (the staining is not sensitive enough); (4) chronicity of the lesions (the patient first presented after 3 months of taking SEC and was then misdiagnosed; he came for a second examination after 2 years). Oral psoriasis was ruled out as there were no marked Munro microabscesses [4].

The differential diagnosis is broad and also includes hairy tongue, hairy leukoplakia, OLP, oral lichenoid reaction (OLR), leukoplakia, friction keratosis, morsication, oral psoriasis, syphilis, and oral lesions associated with coronavirus disease (COVID-19) [22]. Recent data describe OLR, OLP, sialoadenitis, aphthae, EEM, and medication-related osteonecrosis of the jaw (MRONJ) as oral adverse events of SEC [2,4,23,24,25,26,27,28,29,30,31,32,33] (Table 1).

**Table 1:** Oral lesions as oral adverse events of SEC.

Author-Year	A/G	Localisation	Histopathology	Time to Reaction	Time to Resolution	Cessation of SEC	Outcome	Therapy
Thompson (2016) [2]	62/M	Lower lip	Consistent with OLM	1 week	6 weeks	Yes	Recovery	0.1% triamcinolone
Komori (2017) [24]	74/F	Left buccal mucosa	Consistent with OLP	5 months	2 months	Yes	Recovery	Amphotericin B
Capusan (2018) [23]	45/M	Tongue (dorsal/lateral)	Consistent with OLM	8 months	2 months	Yes	Recovery	Intralesional corticosteroids, itraconazole
Benzaquen (2020) [31]	35/F	Soft palate	Consistent with aphthae	5 weeks	3 weeks	No	Recovery	Betamethasone mouthwash
Daye (2021) [28]	33/F	Buccal mucosa	Consistent with OLP	3 months	NR	No	Stabilised	Topical corticosteroid
Picciani (2021) [30]	50/F	Tongue (dorsum/lateral)	Consistent with CHC	2 months	1 month	Yes	Recovery	Topical miconazole gel
Farah (2021) [4]	52/F	Tongue (dorsum)	Consistent with CHC	6 months	6 weeks	No	Recovery	Fluconazole a 50 mg
Pettas (2021) [33]	22/F	Tongue (dorsum), hard palate	Consistent with CHC and pseudomembranous candidiasis	1 week	1 month	No	Recovery	Fluconazole a 100 mg, miconazole gel, CHX 0.2%
Yogarajah (2022) [29]	38/M	Tongue (lateral) and buccal mucosa	Consistent with CHC	6 months	NR	Yes	Recovery	Fluconazole a 100 mg, CHX 0.2%
Fujita (2023) [27]	54/M	Lip and buccal mucosa	Consistent with OLP	1 month	1 month	Yes	Stabilised	Topical miconazole nitrate, topical corticosteroid, topical tacrolimus
Glavina (2025) *	74/M	Tongue (dorsum/lateral)	Consistent with CHC	3 months	6 weeks	Yes	Recovery	Fluconazole a 150 mg, nystatin drops

Abbreviations: SEC: secukinumab; A: age; G: gender; M: male; F: female; OLM: oral lichenoid mucositis; OLP: oral lichen planus; NR: not reported; CHC: chronic hyperplastic candidiasis; CHX: chlorhexidine. * current report.

IL-17 plays an important role in the body’s defence against opportunistic fungal infections [10,34,35]. It also plays an important role in granulopoiesis and in the production of antimicrobial peptides (defensins, histatins, calprotectin, mucin) that destroy the cell membranes of fungi [36,37]. A recent systematic literature review describes the frequency of infections with Candida spp. in 1.7% of patients treated with anti-IL-17A antibodies [20]. There are numerous local (xerostomia, smoking, poor oral hygiene) and systemic (antibiotics, vitamin deficiency, immunosuppression, advanced age, comorbidities) predisposing factors for the development of fungal infections with Candida spp. [38]. New biological therapies/immunotherapies are also predisposing factors for oral candidiasis (CHC, erythematous, pseudomembranous). A dose adjustment of SEC (from 300 mg/150 mg to 75 mg) has led to a significantly lower incidence of Candida infections (50% vs. 10%) [20,39].

Candidiasis often occurs in individuals with an inherited (gene mutations RORγ, RORγt, STAT3, TRAF3IP2, IL-17F, IL-17RA, IL-17RC) or acquired (drug-induced) dysregulation of IL-17 [13]. Oral fungal infections (recurrent) frequently occur in seropositive patients with Human Immunodeficiency Virus (HIV), which is explained by a dysregulation of the immune system—a decrease in the number of Th17 [40]. New biological drugs such as IL-17 inhibitors (SEC dominant) are predisposing factors for the development of candidiasis, with the oral cavity being most frequently affected. This is followed by the genital mucosa, skin, oesophagus, and oropharynx [41,42,43]. The prevalence of oral candidiasis was the same in patients treated with SEC and ixekizumab (1.7%), while it was significantly higher in patients treated with brodalumab (3.8%) and bimekizumab (6.4%) [20,42]. CHC was the most common oral adverse event of SEC (PHD in 45.5%), which is consistent with our case report. The tongue was the most common localisation in 54.5% of cases. The time interval between the start of SEC use and the occurrence of adverse events ranged from one week to eight months, with a median of four months, which is consistent with our case report. The time interval to disappearance or stabilisation of oral lesions ranged from 1 to 2 months, with a median of 1.2 months, which is consistent with our case report. The decision to discontinue SEC therapy was made in 70% of patients, resulting in the disappearance of oral lesions in 81.8% of patients. Oral systemic and local antifungal therapy was the treatment of choice in 72.7% of cases. This is usually sufficient in immunocompetent patients. In immunocompromised patients, other predisposing factors (comorbidities, medications, bad habits) must be controlled and a satisfactory level of oral hygiene maintained [44,45]. Our patient was successfully treated with a combination of local and oral systemic (due to medication-induced immunosuppression) antifungal therapy. In addition, the possibility of treatment with all-trans-retinoic acid (ATRA), a form of vitamin A (retinoid), should be considered in patients treated with IL-17A inhibitors [46]. Vitamin A deficiency is associated with PsO, which predisposes them to fungal infections [38,47]. Routine serological testing for vitamin A/ATRA is recommended in patients treated with IL-17A inhibitors [48].

Dentists and specialists must be familiar with the oral adverse events of the new biological therapies. The description of such rare and atypical cases is important to recognise drug-related or class-related adverse events and to include antifungal therapy as a prophylactic measure in the algorithm for immunocompromised patients. In addition, long-term follow-up is required to monitor response to treatment, recurrence, and known malignancy potential of CHC. Simultaneous monitoring and active discussion between clinical immunology and oral medicine specialists about rare and atypical oral manifestations of biologic therapies are necessary.

## 6. Conclusions

General dentists, oral medicine specialists, dermatologists, and rheumatologists should be familiar with the oral adverse events of SEC on the skin and/or mucous membranes (oral, genital). CHC is an oral potentially malignant disorder (OPMD) that requires long-term follow-up due to the risk of disease recurrence (especially in immunocompromised patients), malignant transformation, and re-biopsy of suspicious lesions. The treatment of SEC patients must be multidisciplinary in order to avoid undesirable consequences and enable a better QoL through timely diagnosis and therapy.

## Data Availability

The data presented can be made available on request.

## Abbreviations

ANAs	Antinuclear Antibodies
AS	Ankylosing Spondylitis
ATRA	All-Trans Retinoic Acid
CHC	Chronic Hyperplastic Candidiasis
CMC	Chronic Mucocutaneous Candidiasis
COVID-19	Coronavirus Disease
cGVHD	Chronic Graft Versus Host Disease
DIF	Direct Immunofluorescence
EBER	Epstein–Barr Virus-encoded small RNA
EBV	Epstein–Barr Virus
EEM	Erythema Exudativum Multiforme
ELISA	Enzyme-Linked Immunosorbent Assay
ENAs	Extractable Nuclear Antigens
FDA	Food and Drug Administration
GERD	Gastro-Oesophageal Reflux Disease
HIV	Human Immunodeficiency Virus
IBD	Inflammatory Bowel Disease
IL-17	Interleukin 17
IL-17A	Interleukin 17-A
IL-23	Interleukin 23
LE	Lupus Erythematosus
MRONJ	Medication-Related Osteonecrosis of the Jaw
MS	Multiple Sclerosis
NHL	Non-Hodgkin Lymphoma
OLDR	Oral Lichenoid Drug Reaction
OLP	Oral Lichen Planus
OLR	Oral Lichenoid Reaction
OPMD	Oral Potentially Malignant Disorder
PAS	Periodic Acid-Schiff
PCI	Percutaneous Coronary Intervention
PHD	Histopathological Diagnosis
PRISMA	Preferred Reporting Items for Systematic Reviews and Meta-Analyses
PsA	Psoriatic Arthritis
PsO	Psoriasis
QoL	Quality of Life
RA	Rheumatoid Arthritis
SEC	Secukinumab
TNF-α	Tumour Necrosis Factor-α
WHO	World Health Organization

## References

[1] Vučićević Boras V., Andabak-Rogulj A., Brailo V., Kraljević Šimunković S., Gabrić D., Vrdoljak D.V. (2015). Adverse drug reactions in the oral cavity. Acta Clin. Croat..

[2] Thompson J.M., Cohen L.M., Yang C.S., Kroumpouzos G. (2016). Severe, ulcerative, lichenoid mucositis associated with secukinumab. JAAD Case Rep..

[3] Novartis (2020). Cosentyx. https://www.cosentyx.com/.

[4] Farah C.S. (2021). Concurrent chronic hyperplastic candidosis and oral lichenoid lesion as adverse events of secukinumab therapy. Aust. Dent. J..

[5] Langley R.G., Elewski B.E., Lebwohl M., Reich K., Griffiths C.E.M., Papp K., Puig L., Nakagawa H., Spelman L., Sigurgeirsson B. (2014). Secukinumab in plaque psoriasis—results of two phase 3 trials. N. Engl. J. Med..

[6] Reich K., Gooderham M., Green L., Bewley A., Zhang Z., Khanskaya I., Day R.M., Goncalves J., Shah K., Piguet V. (2017). The efficacy and safety of apremilast, etanercept and placebo in patients with moderate-to-severe plaque psoriasis: 52-week results from a phase IIIb, randomized, placebo-controlled trial (LIBERATE). J. Eur. Acad. Dermatol. Venereol..

[7] Reich K., Armstrong A.W., Foley P., Song M., Wasfi Y., Randazzo B., Li S., Shen Y.-K., Gordon K.B. (2017). Efficacy and safety of guselkumab, an anti-interleukin-23 monoclonal antibody, compared with adalimumab for the continuous treatment of patients with moderate to severe psoriasis: Results from the phase III, double-blinded, placebo- and active comparator-controlled VOYAGE 1 trial. J. Am. Acad. Dermatol..

[8] de Vries A.C.Q., Thio H.B., de Kort W.J.A., Opmeer B.C., van der Stok H.M., de Jong E.M.G.J., Horvath B., Busschbach J.J.V., Nijsten T.E.C., Spuls P.I. (2017). A prospective randomized controlled trial comparing infliximab and etanercept in patients with moderate-to-severe chronic plaque-type psoriasis: The Psoriasis Infliximab vs. Etanercept Comparison Evaluation (PIECE) study. Br. J Dermatol..

[9] McInnes I.B., Mease P.J., Kirkham B., Kavanaugh A., Ritchlin C.T., Rahman P., van der Heijde D., Landewé R., Conaghan P.G., Gottlieb A.B. (2015). Secukinumab, a human anti-interleukin-17A monoclonal antibody, in patients with psoriatic arthritis (FUTURE 2): A randomised, double-blind, placebo-controlled, phase 3 trial. Lancet.

[10] Conti H.R., Shen F., Nayyar N., Stocum E., Sun J.N., Lindemann M.J., Ho A.W., Hai J.H., Yu J.J., Jung J.W. (2009). Th17 cells and IL-17 receptor signaling are essential for mucosal host defense against oral candidiasis. J. Exp. Med..

[11] Papini M., Natalini Y. (2018). Candida infections in psoriatic patients on anti-IL17 therapy: A case series. J. Dermatolog. Treat..

[12] Kuwabara T., Ishikawa F., Kondo M., Kakiuchi T. (2017). The role of IL-17 and related cytokines in inflammatory autoimmune diseases. Mediat. Inflamm..

[13] Gaffen S.L., Moutsopoulos N.M. (2020). Regulation of host-microbe interactions at oral mucosal barriers by type 17 immunity. Sci. Immunol..

[14] Park H., Li Z., Yang X.O., Chang S.H., Nurieva R., Wang Y.H., Wang Y., Hood L., Zhu Z., Tian Q. (2005). A distinct lineage of CD4 T cells regulates tissue inflammation by producing interleukin 17. Nat. Immunol..

[15] Martin D.A., Towne J.E., Kricorian G., Klekotka P., Gudjonsson J.E., Krueger J.G., Russell C.B. (2013). The emerging role of IL-17 in the pathogenesis of psoriasis: Preclinical and clinical findings. J. Investig. Dermatol..

[16] Kirkham B.W., Kavanaugh A., Reich K. (2014). Interleukin-17A: A unique pathway in immune-mediated diseases: Psoriasis, psoriatic arthritis and rheumatoid arthritis. Immunology.

[17] Chiricozzi A., Guttman-Yassky E., Suárez-Farinas M., Nograles K.E., Tian S., Cardinale I., Chimenti S., Krueger J.G. (2011). Integrative responses to IL-17 and TNF-α in human keratinocytes account for key inflammatory pathogenic circuits in psoriasis. J. Investig. Dermatol..

[18] Baker K.F., Isaacs J.D. (2018). Novel therapies for immune-mediated inflammatory diseases: What can we learn from their use in rheumatoid arthritis, spondyloarthritis, systemic lupus erythematosus, psoriasis, Crohn’s disease and ulcerative colitis?. Ann. Rheum. Dis..

[19] Gaspari A.A., Tyring S. (2015). New and emerging biologic therapies for moderate-to-severe plaque psoriasis: Mechanistic rationales and recent clinical data for IL-17 and IL-23 inhibitors. Dermatol. Ther..

[20] Saunte D.M., Mrowietz U., Puig L., Zachariae C. (2017). Candida infections in patients with psoriasis and psoriatic arthritis treated with interleukin-17 inhibitors and their practical management. Br. J. Dermatol..

[21] Farah C.S., Balasubramaniam R., McCullough M.J. (2019). Contemporary Oral Medicine.

[22] Glavina A., Badrov J., Lukenda M., Džaja K., Biočina-Lukenda D., Lugović-Mihić L. (2024). COVID-19 and oral lesions: 2020-2024 outpatient case series and literature review. Acta Dermatovenerol. Alp. Pannonica Et Adriat..

[23] Capusan T.M., Herrero-Moyano M., Martínez-Mera C.R., Freih-Fraih A.W., Dauden E. (2018). Oral lichenoid reaction in a psoriatic patient treated with secukinumab: A drug-related rather than a class-related adverse event?. JAAD Case Rep..

[24] Komori T., Honda T., Endo Y., Kaku Y., Otsuka A., Kabashima K. (2017). Oral lichen planus associated with candidiasis during secukinumab treatment. J. Dermatol..

[25] Purnell J.C., Williams B.A., Shalin S.C., Wong H.K. (2016). Mucocutaneous findings associated with interleukin (IL)-17 inhibition. JAAD Case Rep..

[26] Hauer L., Moztarzadeh O., Baghalipour N., Gencur J. (2024). Sekucinumab causing medication-realted osteonecrosis of the jaw, in a patient diagnosed with psoriasis and rheumatoid arthritis. Psoriasis.

[27] Fujita Y., Sugai T., Maya Y., Inamura E., Hirano Y., Shimizu S. (2023). Secukinumab-induced oral lichen planus in a psoriatic arthritis patient ameliorated after a switch to risankizumab. J. Dermatol..

[28] Daye M., Temiz S.A., Gümüş S., Kılınç F. (2021). Secukinumab-induced oral lichen planus: A report of case and review of literature. Istanb. Med. J..

[29] Yogarajah S., Mahendran K., Barker J., Setterfield J., Carey B. (2022). Going through a rough patch: Oral adverse effects of secukinumab. Oral Surg..

[30] Picciani B.L.S., Dziedzic A., Werneck J.T., Marinho M.A., Dick T.N.A., Quintanilha N.R., Dias E.P. (2021). Atypical oral candidiasis in a psoriatic patient during targeted immunotherapy with an interleukin 17 inhibitor (secukinumab). BMC Oral Health.

[31] Benzaquen M., Yawalkar N., Feldmeyer L., Borradori L., Schlapbach C. (2020). Herpetiform aphthous ulcerations induced by secukinumab: Report of 2 cases. JAAD Case Rep..

[32] Conforti C., Currado D., Vezzoni R., Corneli P., Bussani R., Navarini L., Magaton-Rizzi G., Fischetti F., Di Meo N., Zalaudek I. (2021). Erythema multiforme induced by secukinumab: Clinical, dermoscopic, and histological features. J. Clin. Rheumatol..

[33] Pettas E., Savva V., Theofilou V.I., Georgaki M., Nikitakis N.G. (2021). Oral *Candida* infection in psoriatic patients treated with IL17A Inhibitors: Report of 3 cases and a comprehensive review of the literature. Diagnostics.

[34] Saunus J.M., Kazoullis A., Farah C.S. (2008). Cellular and molecular mechanisms of resistance to oral Candida albicans infections. Front. Biosci.

[35] Saunus J.M., Wagner S.A., Matias M.A., Hu Y., Zaini Z.M., Farah C.S. (2010). Early activation of the interleukin-23-17 axis in a murine model of oropharyngeal candidiasis. Mol. Oral Microbiol..

[36] Krstic A., Mojsilovic S., Jovcic G., Bugarski D. (2012). The potential of interleukin-17 to mediate hematopoietic response. Immunol. Res..

[37] Jiang L., Fang M., Tao R., Yong X., Wu T. (2020). Recombinant human interleukin 17A enhances the anti-Candida effect of human oral mucosal epithelial cells by inhibiting Candida albicans growth and inducing antimicrobial peptides secretion. J. Oral Pathol. Med..

[38] Picciani B.L.S., Michalski-Santos B., Carneiro S., Sampaio A.L., Avelleira J.C.R., Azulay D.R., Pinto J.M.N., Dias E.P. (2013). Oral candidiasis in patients with psoriasis: Correlation of oral examination and cytopathological evaluation with psoriasis disease severity and treatment. J. Am. Acad. Dermatol..

[39] Blauvelt A., Prinz J.C., Gottlieb A.B., Kingo K., Sofen H., Ruer-Mulard M., Singh V., Pathan R., Papavassilis C., Cooper S. (2015). Secukinumab administration by pre-filled syringe: Efficacy, safety and usability results from a randomized controlled trial in psoriasis (FEATURE). Br. J. Dermatol..

[40] Brenchley J.M., Paiardini M., Knox K.S., Asher A.I., Cervasi B., Asher T.E., Scheinberg P., Price D.A., Hage C.A., Kholi L.M. (2008). Differential Th17 CD4 T-cell depletion in pathogenic and nonpathogenic lentiviral infections. Blood.

[41] Mease P.J., McInnes I.B., Kirkham B., Kavanaugh A., Rahman P., Van Der Heijde D., Landewé R., Nash P., Pricop L., Yuan J. (2015). Secukinumab inhibition of Interleukin-17A in patients with psoriatic arthritis. N. Engl. J. Med..

[42] Gordon K.B., Blauvelt A., Papp K.A., Langley R.G., Luger T., Ohtsuki M., Reich K., Amato D., Ball S.G., Braun D.K. (2016). Phase 3 trials of ixekizumab in moderate-to-severe plaque psoriasis. N. Engl. J. Med..

[43] Bissonnette R., Luger T., Thaçi D., Toth D., Lacombe A., Xia S., Mazur R., Patekar M., Charef P., Milutinovic M. (2018). Secukinumab demonstrates high sustained efficacy and a favourable safety profile in patients with moderate-to-severe psoriasis through 5 years of treatment (SCULPTURE Extension Study). J. Eur. Acad. Dermatol. Venereol..

[44] Pappas P.G., Kauffman C.A., Andes D.R., Clancy C.J., Marr K.A., Ostrosky-Zeichner L., Reboli A.C., Schuster M.G., Vazquez J.A., Walsh T.J. (2016). Clinical practice guideline for the management of candidiasis: 2016 Update by the Infectious Diseases Society of America. Clin. Infect. Dis..

[45] Garcia-Cuesta C., Sarrion-Pérez M.-G., Bagán J.V. (2014). Current treatment of oral candidiasis: A literature review. J. Clin. Exp. Dent..

[46] Campione E., Gaziano R., Marino D., Orlandi A. (2016). Fungistatic activity of all-trans retinoic acid against Aspergillus fumigatus and Candida albicans. Drug Des. Dev. Ther..

[47] Majewski S., Janik P., Langner A., Glinska-Ferenz M., Swietochowska B., Sawicki I. (1989). Decreased levels of vitamin A in serum of patients with psoriasis. Arch. Dermatol. Res..

[48] Campione E., Cosio T., Lanna C., Mazzilli S., Ventura A., Dika E., Gaziano R., Dattola A., Candi E., Bianchi L. (2020). Predictive role of vitamin A serum concentration in psoriatic patients treated with IL-17 inhibitors to prevent skin and systemic fungal infections. J. Pharmacol. Sci..

